# Rapid Proteasomal Degradation of Mutant Proteins Is the Primary Mechanism Leading to Tumorigenesis in Patients With Missense *AIP* Mutations

**DOI:** 10.1210/jc.2016-1307

**Published:** 2016-06-02

**Authors:** Laura C. Hernández-Ramírez, Federico Martucci, Rhodri M. L. Morgan, Giampaolo Trivellin, Daniel Tilley, Nancy Ramos-Guajardo, Donato Iacovazzo, Fulvio D'Acquisto, Chrisostomos Prodromou, Márta Korbonits

**Affiliations:** Centre for Endocrinology (L.C.H.-R., F.M., G.T., D.T., N.R.-G., D.I., M.K.), and Centre for Biochemical Pharmacology (F.D.), William Harvey Research Institute, Barts and The London School of Medicine, Queen Mary University of London, London, EC1M 6BQ, United Kingdom; Genome Damage and Stability Centre (R.M.L.M., C.P.), University of Sussex, Brighton, Falmer, BN1 9RQ, United Kingdom

## Abstract

**Context:**

The pathogenic effect of mutations in the aryl hydrocarbon receptor interacting protein (*AIP*) gene (*AIP*muts) in pituitary adenomas is incompletely understood. We have identified the primary mechanism of loss of function for missense *AIP*muts.

**Objective:**

This study sought to analyze the mechanism/speed of protein turnover of wild-type and missense AIP variants, correlating protein half-life with clinical parameters.

**Design and Setting:**

Half-life and protein–protein interaction experiments and cross-sectional analysis of *AIP*mut positive patients' data were performed in a clinical academic research institution.

**Patients:**

Data were obtained from our cohort of pituitary adenoma patients and literature-reported cases.

**Interventions:**

Protein turnover of endogenous AIP in two cell lines and fifteen AIP variants overexpressed in HEK293 cells was analyzed via cycloheximide chase and proteasome inhibition. Glutathione-S-transferase pull-down and quantitative mass spectrometry identified proteins involved in AIP degradation; results were confirmed by coimmunoprecipitation and gene knockdown. Relevant clinical data was collected.

**Main Outcome Measures:**

Half-life of wild-type and mutant AIP proteins and its correlation with clinical parameters.

**Results:**

Endogenous AIP half-life was similar in HEK293 and lymphoblastoid cells (43.5 and 32.7 h). AIP variants were divided into stable proteins (median, 77.7 h; interquartile range [IQR], 60.7–92.9 h), and those with short (median, 27 h; IQR, 21.6–28.7 h) or very short (median, 7.7 h; IQR, 5.6–10.5 h) half-life; proteasomal inhibition rescued the rapid degradation of mutant proteins. The experimental half-life significantly correlated with age at diagnosis of acromegaly/gigantism (r = 0.411*; P* = .002). The FBXO3-containing SKP1–CUL1–F-box protein complex was identified as the E3 ubiquitin-ligase recognizing AIP.

**Conclusions:**

AIP is a stable protein, driven to ubiquitination by the SKP1–CUL1–F-box protein complex. Enhanced proteasomal degradation is a novel pathogenic mechanism for *AIP*muts, with direct implications for the phenotype.

## Abstract (precis)

We determined that protein instability, leading to shortened protein half-life, is a novel pathogenic mechanism for mutations in the AIP gene with clinical significance for pituitary adenoma patients.

Loss-of-function mutations of the tumor suppressor aryl hydrocarbon receptor interacting protein (*AIP*) gene (*AIP*muts) constitute the genetic basis of a subset of familial isolated pituitary adenomas and sporadic cases of pituitary adenomas in young patients (1–3). Most of the clinically relevant *AIP*muts result in partial loss of the tetratricopeptide (TPR) repeats and the final α-7 helix of the C-terminal TPR domain of the protein (truncating mutations) (4), expectedly leading to unstable or nonexistent proteins (5). Predicting, however, the pathogenic mechanisms of the missense variants can be challenging. The amino acid sequence of the AIP TPR motifs is important to ensure a correct interaction among residues in neighboring alpha helices, as well as adequate protein folding (6). The conserved amino acids located in the pocket between the TPRs 2 and 3 are essential for the interaction of AIP with HSP90 (5). Missense mutations affecting residues involved in the folding of the TPR domain could result in misfolded proteins, which are usually unstable, and in turn may lead to a loss of their ability to interact with other proteins (7).

Various approaches are available to determine whether nontruncating *AIP*muts are indeed pathogenic or not. Evaluating the segregation of the variant with the phenotype in large pedigrees with full medical history (8) would be ideal, although incomplete penetrance and scarcity of large families render this approach difficult. The finding of loss of heterozygosity (LOH) in the tumoral tissue could support the pathogenic role of a variant (9), although 11q13 loss is commonly described in pituitary adenomas (10, 11). The occurrence of a variant in hotspot residues as a result of independent mutational events supports its pathogenicity (12), although only few hotspots (amino acids 81, 271, and 304) exist in AIP. In vitro functional studies could be useful (8), but the most appropriate assay to evaluate *AIP* variants has not yet been established.

In contrast, amino acid substitutions altering the structure, folding or stability of the protein, could lack an obvious functional effect in in vitro studies. In combination with in silico models (13), the evaluation of protein stability could help to determine whether the mutant proteins are actually produced and if their turnover is normal, regardless of their function (14). Protein half-life depends on the rate of protein degradation and is usually decreased in truncated or misfolded proteins (15). We aimed to analyze the mechanism and speed of protein turnover of wild-type (WT) AIP and AIP missense variants, correlating the protein half-life with the phenotype, using reduced in vitro protein stability as a parameter suggesting loss or compromised function. Our data show correlation between in vitro half-life data and the clinical parameters of the particular patients harboring missense variants.

## Materials and Methods

The detailed experimental procedures and statistical analyses are provided in Supplemental Methods. In brief, in AIP half-life experiments, HEK293 and EBV-immortalized cells (derived from a control subject [EBV-LC-AIP_WT] and a patient with a heterozygous *AIP*mut [EBV-LC-AIP_p.R304*]) were treated with cycloheximide (CHX, 100 μg/mL) and total protein was extracted at various time points for Western blot (WB). RNA stability was analyzed using RT-qPCR. Fifteen *AIP* variant plasmids (obtained by site-directed mutagenesis, Tables 1 and 2) were transfected into HEK293 cells for half-life experiments (CHX, 20 μg/mL, Table 2), in the presence or absence of the proteasome inhibitor MG-132 (20 μM). Genetic and clinical data were collected from 100 pituitary adenoma patients [60 from our cohort (4) and 40 cases reported in the literature] carrying the missense *AIP* variants as well as the nonsense mutation we have studied here. AIP interacting partners were identified in a pull-down assay from rat GH-secreting GH3 cells using glutathione-S-transferase-fused WT and mutant AIP synthetic proteins followed by quantitative mass spectrometry. Coimmunoprecipitation and siRNA knockdown (KD) of *FBXO3* were used to study the role of FBXO3 in AIP degradation. Half-life experiments were analyzed using a one-phase decay equation, and the degradation speed (K) was compared between each mutant protein and the WT protein using the extra sum-of-squares F test. Correlation between half-life and clinical features was analyzed using data derived from studies listed in Table 3 using the Spearman R test. For other analyses, Kruskal-Wallis, one-way ANOVA, and linear regression tests were used as appropriate, and significance was taken as *P* < .05.

**Table 1.** Missense Variants Included in the Study

Variant	Location in Protein	Pathogenic	Effect on Protein Structure
c.47G>A (p.R16H)	N terminus	No	No effect, residue out in solvent
c.145G>A (p.V49M)	PPIase domain	Unlikely	Out in solvent, strange for a hydrophobic change. Probably involved in interactions
c.509T>C (p.M170T)	Between PPIase and TPR1 domains	Likely	No structure available^a^
c.562C>T (p.R188W)	TPR1 domain	Likely	No prediction available
c.713G>A (p.C238Y)	TPR2 domain	Yes	Mutation causes severe steric clashes, disrupts packing of hydrophobic core (5)
c.760 T>C (p.C254R)	TPR2 domain	Likely	No prediction available
c.762C>G (p.C254W)	TPR2 domain	Likely	No prediction available
c.769A>G (p.I257V)	TPR2 domain	Likely	Disrupts packing of hydrophobic core (5)
c.811C>T (p.R271W)	TPR3 domain	Yes	Involved in packing with numerous polar interactions. Tryptophan at this position is likely to be disruptive (5)
c.827C>T (p.A276V)	TPR3 domain	Unlikely	Involved in packing, some steric clash with a valine residue, but difficult to draw a definitive conclusion on the effect
c.871G>A (p.V291M)	TPR3 domain	Likely	Disrupts packing of hydrophobic core (forms base of hydrophobic pocket interacting with bound peptide) (5)
c.896C>T (p.A299V)	TPR3 domain	Unlikely	At start of C-terminal α-7 helix and may disrupt some small degree of packing with L292 (5)
c.911G>A (p.R304Q)	TPR3 domain	Yes	Not involved in packing, but probably required for client protein interaction (5)
c.974G>A (p.R325Q)	C-terminal α-helix	Likely	Part of the C-terminal α-7 helix, may affect client protein binding (5)

a This residue is neither included in the nuclear magnetic resonance spectroscopy structure of the peptidylprolyl cis-trans isomerase (PPIase) domain, nor in the crystal structure of the TPR domain.

**Table 2.** Half-life of Overexpressed AIP (WT and Variants) in HEK293 Cells

Variant	Location in Protein	Half-Life Transfected Protein, h	Degradation Speed, K	*P* Value, K Versus WT K
c.47G>A (p.R16H)	N terminus	94.2	0.0074	.0512
c.145G>A (p.V49M)	PPIase domain	27.0	0.0257	**.0359**
c.509T>C (p.M170T)	Between PPIase and TPR1 domains	66.6	0.0104	.2931
c.562C>T (p.R188W)	TPR1 domain	21.0	0.0331	**<.0001**
c.713G>A (p.C238Y)	TPR2 domain	5.5	0.1270	**<.0001**
c.760 T>C (p.C254R)	TPR2 domain	4.7	0.1467	**<.0001**
c.762C>G (p.C254W)	TPR2 domain	8.4	0.0830	**<.0001**
c.769A>G (p.I257V)	TPR2 domain	28.7	0.0241	**.0222**
c.811C>T (p.R271W)	TPR3 domain	8.2	0.0849	**<.0001**
c.827C>T (p.A276V)	TPR3 domain	7.2	0.0957	**<.0001**
c.871G>A (p.V291M)	TPR3 domain	11.2	0.0621	**<.0001**
c.896C>T (p.A299V)	TPR3 domain	21.6	0.0321	**.0002**
c.911G>A (p.R304Q)	TPR3 domain	58.7	0.0118	.5644
c.910C>T (p.R304*)	C-terminal α-helix	5.9	0.1183	**<.0001**
c.974G>A (p.R325Q)	C-terminal α-helix	88.8	0.0078	.0948

Bold characters in the last column denote statistically significant values.

**Table 3.** Missense *AIP* Variants and nonsense p.R304* variant in Pituitary Adenoma Patients: Clinical Features

Variant	No. of Cases			Clinical Presentation	Sex, M/F	Age at Onset, Median (IQR)	Age at Diagnosis, Median (IQR)	Macro/Micro, No.	References
	Our Cases	Other Cases	Total						
c.509T>C (p.M170T)	0	1	1	Simplex	1/0	NA	32	NA	(21)
c.562C>T (p.R188W)	1	0	1	Simplex	1/0	10	12	1/0	Current report
c.713G>A (p.C238Y)	3	0	3	Familial	3/0	20 (18–22)	21 (19–23)	2/0	(3,4,43)
c.760T>C (p.C254R)	1	0	1	Simplex	0/1	14	17	1/0	Current report
c.762C>G (p.C254W)	2	0	2	Familial	1/1	26 (21–31)	28 (23–33)	1/0	Current report
c.769A>G (p.I257V)	0	1	1	Simplex	1/0	NA	NA	NA	(44)
c.811C>T (p.R271W)	1	8	9	Both	7/2	15.5 (12.8–18.3)	22 (14–28)	9/0	(2,4,25,45,46)
c.871G>A (p.V291M)	0	1	1	Simplex	0/1	NA	30	NA	(47)
c.911G>A (p.R304Q)	13	8	21	Both	6/13^#^	27 (22–37)	36 (24.3–39.5)	14/3	(3,4,21,24,25,46–50)
c.910C>T (p.R304*)	39	18	57	Both	31/26	17 (15–25.8)	19.5 (17–28.5)	43/5	(3,4,21,25,46,49–53)
c.974G>A (p.R325Q)	0	3	3	Both	1/2	16 (7–33)	18 (16–35)	3/0	(21,54)
Total	60	40	100		52/46	18 (15–27)	23 (17–32)	73/8	

Abbreviations: Macro/micro: macroadenomas/microadenomas; NA, not applicable.

Age at onset and at diagnosis is expressed in years.

# Gender was not available in two cases.

## Results

### The half-life of endogenous AIP is constant in different cell lines

**Figure 1.**
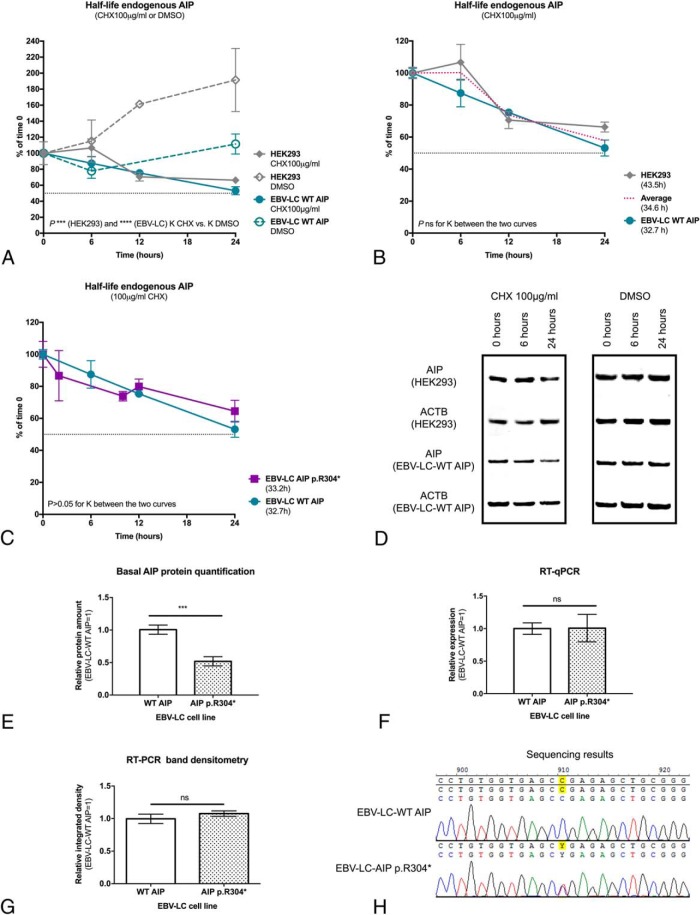
Half-life of endogenous AIP in different cell lines. A, The measured AIP half-life in CHX-treated HEK293 and EBV-LC-AIP_WT cells was significantly shorter compared with DMSO controls in HEK293 and EBV-LC-AIP_WT cells (*P* < .0001 for both cell lines), confirming that the findings under these experimental conditions are due to the effect of CHX. ACTB, beta-actin. B, AIP half-life in HEK293 cells was not significantly different than the half-life in EBV-LC-AIP_WT. C, AIP half-life was almost identical in EBV-LC-AIP_WT and EBV-LC-AIP_p.304* cells, when considering band densitometry for the normal protein. D, In the half-life experiment using the EBV-LC-AIP_p.304* cells, the band for the p.R304* mutant (expected mass, 34.5 kDa) was not detected. In the representative WB images, the top panels are for the experiments with CHX and DMSO in HEK293 cells and the bottom panels are for EBV-LC-AIP_WT cells. WB bands for AIP (37.6 kDa) and the loading control beta-actin (ACTB) (41.7 kDa) are shown in each case. E, Protein quantification in basal conditions demonstrates a reduced level of the normal AIP protein in the EBV-LC-AIP_p.R304* cells, compared with the WT cell line. The differential expression of AIP observed in the two cell lines should be due to posttranslational regulation, the basal *AIP* mRNA levels are not different between the two cell lines, quantified by (F) RT-qPCR and (G) semiquantitative RT-PCR band densitometry. H, In concordance with these findings, the mutant cell line displays heterozygosity for *AIP* c.910C>T at the cDNA level.

Under the experimental conditions used, the half-life of endogenous AIP in CHX-treated HEK293 and EBV-LC-AIP_WT cells was significantly shorter compared with the dimethylsulfoxide (DMSO) controls (*P* < .0001 for both cell lines, Figure 1A). The half-life of AIP was not significantly different between the two cell lines (HEK293, 43.5 h vs EBV-LC-AIP_WT, 32.7 h; *P* = .2537; Figure 1B).

AIP half-life was practically identical between EBV-LC-AIP_WT and EBV-LC-AIP_p.R304* cells (33.2 h for AIP_p.R304*; Figure 1C). Assuming biallelic expression, bands of 37.6 kDa for WT AIP and 34.5 kDa for the truncated protein AIP p.R304* (as calculated by the Expasy Compute pI/Mw tool) (16) were expected in the WB images for the EBV-LC-AIP_p.R304* cells. However, the band for the mutant protein was not detected (Figure 1D), indicating that the mutant protein is either not expressed or very rapidly degraded. The basal expression of the WT AIP protein was significantly lower in the EBV-LC-AIP_p.304* cells (49 ± 17% of the level in the AIP_WT cells; *P* = .0006; Figure 1E) in agreement with the above finding for the p.R304* mutant. Nevertheless, the finding that the half-life of the WT protein is normal in these mutant cells excludes the possibility of a dominant-negative effect of the heterozygous nonsense mutation on the stability of WT AIP.

### The absence of the mutant AIP p.R304* protein is due to posttranslational regulation

The basal expression of *AIP* was not significantly different between EBV-LC-AIP_WT and EBV-LC-AIP_p.R304* cells, measured by qPCR (WT; 1.1; interquartile range, [IQR], 0.6–1.3 vs p.R304*, 0.6; IQR, 0.5–2.2; *P* = .7; Figure 1F) and conventional RT-PCR band densitometry (WT, 0.9; IQR, 0.9–1.1; vs p.R304*, 1; IQR, 1–1.2; Figure 1G). Furthermore, the presence of the mutant allele was confirmed by cDNA sequencing (Figure 1H). These results suggest that both the mutant and the WT allele are equally expressed at the mRNA level; therefore, the lack of expression of the mutant protein should be due to posttranslational regulation.

### Variable half-life of overexpressed WT AIP and missense variants

**Figure 2.**
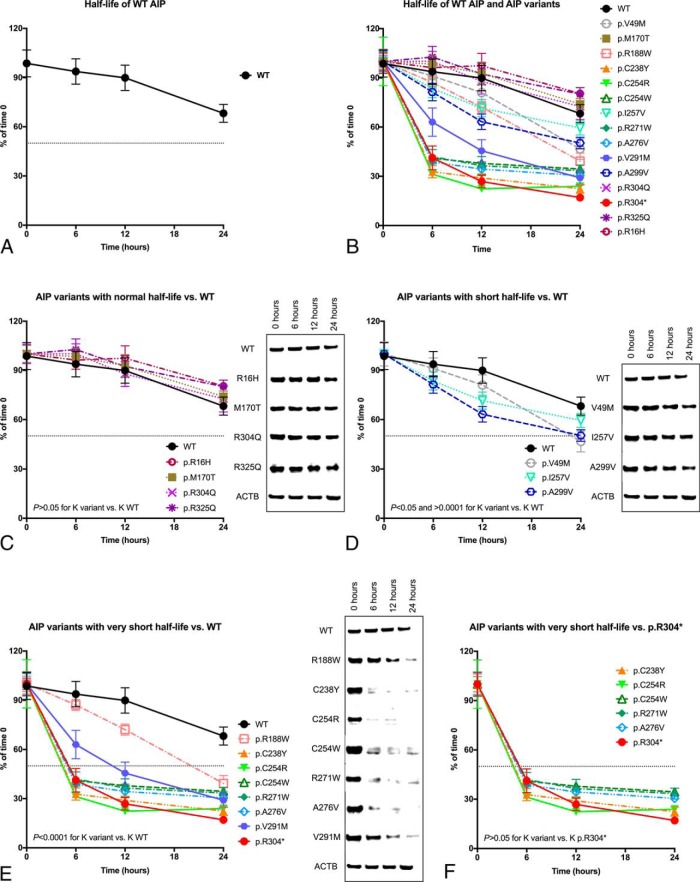
Half-life of WT AIP and variants, overexpressed in HEK293 cells. A, Half-life curve for WT AIP. B, Half-life curves for all the variants studied, compared with WT AIP. Comparisons with the WT protein were established by means of the degradation speed (K) calculated from each half-life curve. C, Half-life curves for AIP variants with normal half-life (p.R16H, p.M170T, p.R304Q, and p.R325Q) and representative WB images, compared with WT AIP. D, Half-life curves for AIP variants with “short” half-life (p.V49M, p.I257V, p.A299V) and representative WB images, compared with WT AIP. E, Half-life curves for missense AIP variants with “very short” half-life (p.R188W, p.C238Y, p.C254R, p.C254W, p.R271W, p.A276V, p.V291M) and representative WB images, compared with the WT protein. F, Half-life curves for variants with half-life similar to the nonsense variant p.R304* (p.C238Y, p.C254R, p.C254W, p.R271W, p.A276V) and representative WB images, compared with AIP p.R304*. Myc-AIP, 39 kDa; Myc-AIP p.R304*, 35.8 kDa; ACTB, 41.7 kDa. ACTB loading control shown for the WT experiment in each case.

We compared the half-life of the overexpressed WT AIP protein in HEK293 cells (48 h; Figure 2A) with that of several AIP variants (Figure 2B and Table 2). The variants p.R16H, p.M170T, p.R304Q, and p.R325Q behaved as stable proteins, with half-life similar to that of the WT protein (median, 77.7 h; IQR, 60.7–92.9; Figure 2C). The rest of the missense variants displayed shortened half-life when compared with the WT protein. A group of mutant proteins (p.V49M, p.I257V, p.A299V) was considered to have a “short” half-life (27 h; IQR, 21.6–28.7), because the difference between their degradation speed (K) and that of the WT protein resulted in *P* < .05 but *P* > 0.0001 (Figure 2D). Variants whose K displayed maximum statistical significance (*P* < .0001) when compared with the WT protein were considered to have “very short” half-life: p.R188W, p.C238Y, p.C254R, p.C254W, p.R271W, p.A276V, p.V291M (7.7 h; IQR, 5.6–10.5; Figure 2E). The variants with the shortest half-lives (p.C238Y and p.C254R) were located in the second TPR motif of the protein.

The pathogenic nonsense AIP variant p.R304* had a “very short” half-life (5.9 h), in concordance with the proposed unstable behavior of this variant (3, 17). Interestingly, all the missense variants with “very short” half-life, except p.R188W and p.V291M, had degradation speeds comparable with that of the p.R304* variant (Figure 2F and Supplemental Table 1). Also similar to p.R304*, their degradation curves were characterized by a fast decay of the protein in the first 6 hours, followed by a plateau. In contrast, the curves for p.R188W and p.V291M displayed gradual decay throughout the duration of the experiment, similar in shape to that of the variants with “short” half-life.

### Short-lived AIP variants are partially rescued by proteasome inhibition

**Figure 3.**
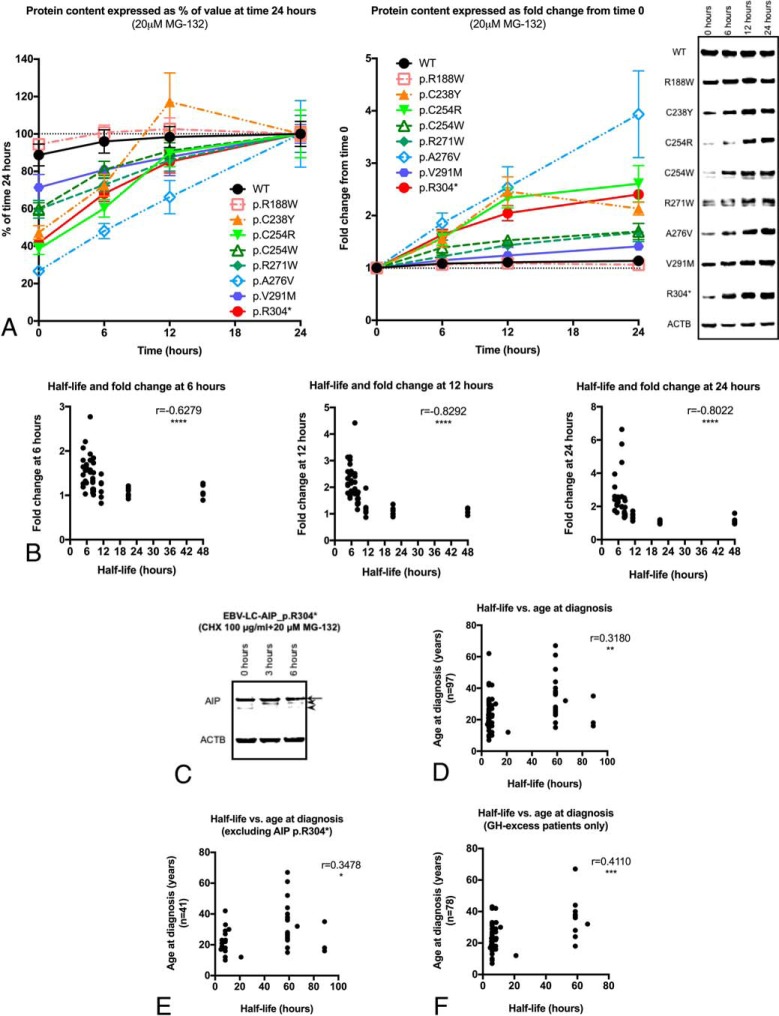
Blocking of proteasomal degradation with MG-132 (“rescue experiments”) and implications of AIP half-life for the phenotype in pituitary adenoma patients. A, Curves of protein contents for the experiments with the variants and WT protein, expressed as percentages (level at the 24-h time point was considered as 100% for each protein) or as fold change from time 0 (time 0 = 1) and representative WB images. The AIP variants p.R188W (fold change at 24 h, 1.1; global *P* = .5080) and p.V291M (fold change at 24 h, 1.4; global *P* = .1263) were stable. In contrast, levels of the other variants studied displayed a significant rise in response to MG-132: p.C238Y (fold change, 1.6 [*P* = .0403]; 2.5 [*P* = .0170]; and 2.1 [*P* = .0015] at 6, 12, and 24 h, respectively), p.C254R (fold change, 1.6 [*P* = .0087]; 2.3 [*P* = .0008]; and 2.6 [*P* = .0094]), p.C254W (fold change, 1.4 [*P* = .0910]; 1.5 [*P* = .0006]; and 1.7 [*P* = .0087]), p.R271W (fold change, 1.4 [*P* = .1796]; 1.5 [*P* = .0229]; and 1.7 [*P* = .0676]), p.A276V (fold change, 1.9 [*P* = .0072]; 2.5 [*P* = .0120]; and 3.9 [*P* = .0225]) and p.R304* (fold change, 1.6 [*P* = .0055]; 2 [*P* = .0041]; and 2.4 [*P* < .0001]). Myc-AIP, 39 kDa; Myc-AIP p.R304*, 35.8 kDa; ACTB, 41.7 kDa. ACTB loading control shown for the WT experiment in each case. B, Correlation between half-life and fold change after MG-132 treatment at 6, 12, and 24 h. A significant indirect correlation was found at the three time points, indicating proteins with shorter half-lives responded better to proteasome inhibition. C, MG-132 treatment of EBV-LC-AIP_p.R304* cells. In the AIP WB (using 40 μg of total protein) the strongest band (arrow) corresponds to the WT protein (37.6 kDa). Two faint extra bands can be observed: a first band that is absent at time 0 and appears after the treatment with MG-132 (top arrowhead) and a second, lower band (bottom arrowhead), which is more evident at time 0. Although both bands could correspond to degradation products, the heaviest one could possibly be given by a small amount of truncated protein, appearing after proteasomal degradation is inhibited (expected size, 34.5 kDa). Loading control: ACTB (41.7 kDa). D, The half-life of the studied AIP variants directly correlated with the age at diagnosis, (E) even when excluding patients with the p.R304* mutant; (F) the significance increased when considering only patients with acromegaly or gigantism.

Stable WT AIP protein levels were found under proteasome inhibition with MG-132, with minimal, but not significant increase at 0, 6, 12, and 24 hours of treatment (fold change at 24 h: 1.1; global *P* = .3592; Figure 3A). Likewise, levels of the AIP variants p.R188W and p.V291M remained stable, whereas the levels of the rest of the variants studied, including the p.R304* nonsense variant, significantly increased in response to MG-132.

Pooling all these results together, a significant negative correlation was found between protein half-life and protein levels at 6 (r = −0.6279), 12 (r = −0.8292), and 24 hours (r = −0.8022) of MG-132 treatment (*P* < .0001 for all the time points; Figure 3B), meaning that proteins with shorter half-life displayed a more-dramatic response to MG-132. These findings confirm that WT AIP is a stable protein, whose degradation is driven by the ubiquitin proteasome system (UPS), and that enhanced proteasomal degradation is responsible for the reduced half-life shown by some missense variants.

After the treatment of EBV-LC-AIP_p.R304* cells with the proteasome inhibitor MG-132 for 3 or 6 hours, the WB images displayed an additional band with a slightly faster migration, compared with the WT protein (Figure 3C). A third extra band, below the second one, was present at 0 hours and disappeared after treatment with the proteasome inhibitor. Although both of these bands could represent degradation products, one of them could account for AIP p.R304*, considering its apparent molecular weight.

### Correlating protein half-life and phenotype

Clinical data from 100 pituitary adenoma patients carrying the missense *AIP* variants and the nonsense variant included in the half-life experiments were collected (Table 3). The variants c.47G>A (p.R16H), c.145G>A (p.V49M), and c.896C>T (p.A299V) were not included in this clinical analysis, given that they are not considered pathogenic based on previous clinical and functional evaluations. The variant c.827C>T (p.A276V) was also excluded, given that it has never been found in pituitary adenoma patients (detected only in a screening for *AIP* single-nucleotide polymorphisms in DNA samples from different populations) (18).

A direct correlation was found between age at diagnosis and protein half-life (r = 0.3180; *P* = .0015; Figure 3D). This remained significant after excluding patients carrying the p.R304* nonsense variant (r = 0.3478; *P* = .0259; Figure 3E). As *AIP*muts are mainly prevalent among patients with GH excess (acromegaly or gigantism), we analyzed this group separately, finding also a significant correlation between age at diagnosis and protein half-life (r = 0.411; *P* = .002; Figure 3F). The proportion of patients with macroadenomas (vs microadenomas) was similar for the variants with “very short” half-life compared with other variants (91.8 vs 85%; *P* = .4007).

### The SKP1–CUL1–F-box protein (SCF) E3 ubiquitin-ligase complex containing the FBXO3 regulates AIP half-life

Two members of the SCF E3 ubiquitin-ligase complex were detected by our pull-down experiments: the F-box containing protein FBXO3 and SKP1, with differential representation among different pull-down experiments (Supplemental Figure 1A). Validation by coimmunoprecipitation with WT AIP was positive only for FBXO3 (Supplemental Figure 1B); however, if AIP is directed to ubiquitination by the SCF complex, it is expected to interact with FBXO3 and not with SKP1. In concordance with this, stable endogenous AIP levels were found when *FBXO3* was partially silenced by KD (Supplemental Figure 2), whereas *FBXO3* overexpression resulted in a curve resembling that of the untransfected cells. The scrambled siRNA control resulted in slightly reduced K, whereas the rescue experiment rendered results similar to those of the KD, although no statistically significant differences were found. Nevertheless, the K obtained in these experiments was directly influenced by the level of *FBXO3* expression, as shown by linear regression (r^2^ = 0.7915; *P* = < .0001). These results suggest that AIP is targeted and bound by the SCF E3 ubiquitin-ligase complex, via the F-box-containing protein, FBXO3, to be degraded by the UPS. The differential affinity in the pull-down experiment suggests that the p.R304* mutant is more prone to be ubiquitinated than the WT protein.

## Discussion

We measured the half-life of 15 AIP variants and established that most of the pathogenic missense mutants have a short or very short half-life. Furthermore, the half-life of mutant proteins correlated with age at diagnosis of acromegaly/gigantism in the patients harboring these mutations.

The maintenance of intracellular proteostasis relies on the correct functioning of complex cotranslational and posttranslational mechanisms to determine the fate of proteins (repair vs degradation), a process often referred to as “protein quality control” (19). Protein and mRNA half-lives are strictly regulated: usually genes with constitutive cell functions have both stable mRNAs and proteins, whereas transcription factors, signaling molecules, chromatin-modifying enzymes, and genes with cell cycle–specific functions tend to have unstable mRNAs and proteins; abundant and highly structured proteins are more stable than the less-abundant and less-structured ones (20). Not surprisingly, under the experimental conditions used, the abundantly expressed cochaperone AIP behaved as a stable protein, with a half-life of 32.7–43.5 hours. The half-life of endogenous human AIP (32.7 h) was very similar to the reported half-life for the mouse AIP (30.4 h), measured by stable isotope labeling by amino acids in cell culture in NIH 3T3 cells (20).

For most of the AIP variants we studied, the pathogenicity predictions matched the half-life results (ie, pathogenic variants had very short half-life). However, given that the pathogenic mechanisms of variants are likely to be variable, and there may not be a single in vitro method to identify pathogenicity of all the missense mutants, we observed some discrepancies. Only one of the four normal half-life variants is currently considered nonpathogenic (p.R16H), whereas the other three (p.M170T, p.R304Q, p.R325Q) were suggested to be pathogenic. The variant p.M170T has only been reported in one patient with young-onset acromegaly (21), there is no functional data published and its minor allele frequency (MAF) is not available in public databases. Besides the in silico prediction and clinical data, no functional data are available for p.R325Q (MAF 0.00006) (22). For the much more common variant, p.R304Q (21 patients and MAF 0.0007 (23) and 0.0015 (22) in two databases, including a homozygous subject), although clinical data strongly supports a pathogenic role, most of the experimental results are against a negative effect on protein function (24, 25). If these three variants are indeed pathogenic, the mechanism should be related to loss of a function that is independent of protein stability. This is consistent with our previous hypothesis that lack of client protein interaction is sufficient for pituitary adenoma predisposition (5).

Our “short” half-life group (defined based on arbitrary cutoffs for statistical significance) included three variants with different predicted effects. The AIP variant p.A299V [MAF 0.001 (23, 26) and 0.0004 (22)] is considered unlikely pathogenic based on clinical data together with the results of different functional studies (3, 24, 25). In contrast, a damaging structural effect has been predicted for p.I257V, given that this variant affects a well-conserved amino acid in the second TPR domain of AIP (5, 27), matching the clinical data, and therefore confirming its pathogenic effect. In the case of p.V49M [MAF 0.0002 (22, 23)] the change of a hydrophobic (V) for a polar (M) amino acid could possibly disrupt the folding of the PPIase domain. Our half-life data support a pathogenic role for this variant, detected in a simplex gigantism case, with no LOH in the tumor (28).

The endogenous mutant protein could not be detected in EBV-LC-AIP_p.R304* cells, but it apparently became visible following proteasome inhibitor treatment, supporting the findings of very unstable behavior for this C-terminally truncated protein. Our experiments demonstrate that the absence of detectable protein is due to a posttranslational mechanism (proteasomal degradation), given that nonsense-mediated mRNA decay was ruled out. Protein interaction studies have shown a partial loss of interaction with HSP90 and HSPA8 for this mutant (unpublished data by our group). As these chaperones facilitate the correct folding of their partner proteins, loss of the AIP C-terminal interaction site could disrupt the folding of its TPR motifs. This is supported by the fact that the effect of this nonsense mutation on protein half-life was similar to that of the missense mutations affecting the TPR repeats 2 and 3. In addition, misfolding and the loss of interactions with certain partners could make these proteins more prone to ubiquitination and proteasomal degradation. Some AIP TPR mutants that are unable to bind HSP90 or to assemble AHR/HSP90 complexes have been described to have a normal half-life. This suggests that AIP stability could be independent of HSP90 binding (29) and other partners could stabilize the protein. Conversely, if a short half-life is translated into an almost absent protein under physiological conditions, it could be assumed that the endogenously expressed missense variants with “short” or “very short” half-life would be present also at low or very low levels in the cells.

As *AIP*muts have so far always been found in the heterozygous state, a possible dominant-negative effect of the mutant protein on normal AIP could be considered, and our experiments provide data to address this issue. In this regard, the half-life experiments have two different implications. Firstly, the fact the half-life of the WT AIP is normal in the presence of the p.R304* unstable variant rules out the possibility of a dominant-negative effect of this mutant protein, an effect that accounts, for example, for the loss of tumor-suppressor function of TP53 (30). Secondly, considering that the expression of *AIP* is apparently biallelic, the expression of both alleles might be necessary for AIP to perform its tumor suppressor functions under basal or stimulated conditions, and the reduction of total AIP expression could be insufficient for an antitumorigenic effect in the pituitary gland (haploinsufficiency) (31). Our study did not investigate whether the apparently pathogenic variants with normal half-life behave in a similar way, or if in those cases the proteins are expressed at normal levels, but interfere with the stability or function of the normal protein (ie, if they have dominant-negative effect).

The half-life of the AIP variants studied directly correlated with the age at diagnosis in pituitary adenoma patients carrying those mutations. This finding is important and clinically significant, and contributes to the validation of the half-life assay as a method for determining the pathogenicity of *AIP* missense variants. In addition, it suggests that the age at disease onset in the setting of *AIP*muts can be determined by the amount of functional protein available. The application of a protein stability assay as a means to evaluate the pathogenic effect of missense variants has also been proposed for *MEN1* (32, 33), and for *PRKAR1A* (34), two genes implicated in endocrine tumor-predisposing syndromes. Nevertheless, protein stability is a parameter that cannot be evaluated with routine diagnostic histopathological procedures. Given that AIP is a stable protein, abundantly expressed in the pituitary gland, the static image of the tissue obtained by immunohistochemistry might not be sensitive enough to detect changes in the AIP protein levels due to enhanced degradation, especially in tumors with no LOH.

Proteasomal degradation of proteins is facilitated by E3 ubiquitin ligases. The SCF, a multiprotein complex, is the prototypical cullin-really interesting new gene (RING) E3 ubiquitin-ligase, consisting of the RING-containing enzyme RBX1, the cullin scaffold CUL1, the adaptor protein SKP1 and a variable F-box containing protein, the latter defining the specificity of the protein degradation via the UPS (35, 36). Our findings suggest that AIP is specifically targeted by FBXO3 to be bound by the SCF E3 ubiquitin-ligase complex and delivered to UPS. Although KD of *FBXO3* resulted in prolonged AIP half-life, this effect was not statistically significant. This could be explained by the influence of more than one E3 ubiquitin ligase on AIP targeting, as it occurs for the tumor suppressor TP53 (37).

Dysfunction of the UPS pathway is related to a variety of human disorders, either due to reduced or increased ubiquitination of target proteins (38), including corticotroph adenomas (39–41). Different steps of the UPS represent targets for recently developed drugs for the treatment of human neoplasms and other conditions (38). The proteasome inhibitor MG-132 acts at the chymotrypsin-like site of the proteasome (also at the caspase-like site at high concentrations), in a similar way to the clinically available proteasome inhibitor bortezomib (PS-341) (42). Proteasome inhibitors could theoretically restore the levels of mutant AIP, as it has recently been proposed also for menin (32), and could therefore have a role in the treatment of aggressive pituitary adenomas. However, the effects of these drugs include regulation of multiple pathways implicated in tumorigenesis, and therefore this possible application should be explored in depth in preclinical studies.

### Conclusions

Human AIP is a stable protein whose degradation is driven by ubiquitination and proteasomal degradation mediated by the FBXO3-containing SCF E3 ubiquitin-ligase complex. Most of the clinically relevant missense *AIP* variants tested, especially those affecting the TPR domain of the protein, as well as the nonsense mutant p.R304*, displayed reduced protein half-life due to enhanced proteasomal degradation. These unstable proteins can be partially rescued by proteasome inhibition. A direct significant correlation between protein half-life and age at diagnosis in *AIP*mut-positive pituitary patients was found. The coexistence of an unstable AIP mutant does not seem to affect the half-life of the WT AIP protein. We describe here a novel pathogenic mechanism of reduced half-life of AIP mutants with close correlation with clinical parameters.
